# Supplementary material for: COVID-19 related immunization disruptions in Rajasthan, India: A retrospective observational study
Source: Vaccine. 2021 Jul 13;39(31):4343–50. doi: 10.1016/j.vaccine.2021.06.022 (PMC8196298; doi:10.1016/j.vaccine.2021.06.022)
Supplement: Supplementary data 1 [file mmc1.pdf]

COVID-19 Related Immunization Disruptions in Rajasthan, India:
A Retrospective Observational Study
Supplementary Materials

Authors: Radhika Jain, Ambika Chopra, Camille Falézan, Mustufa Patel, Pascaline Dupas
Correspondence to: rjain19@stanford.edu

Table of Contents

A. HMIS Analysis 2
Figure S1: Monthly HMIS Trends of Fully Immunized Children Aged 9-11 Months in Rajasthan 3
Figure S2: HMIS Changes in Monthly Fully Immunized Children Aged 9-11 Months in 2020 3
Table S1: HMIS Monthly Immunization Trends in Rajasthan and India 4
B. Survey Analysis 4
Table S2: Survey Status 4
Table S3: Household Characteristics by Survey Status 5
Table S4: Characteristics of Children With and Without an Immunization Card 5
Table S5: Child Characteristics Across Exposure Groups 5
Table S6: Measles1 Immunization at or before 9 months and between 10-12 months by Lockdown Exposure 6
Figure S3: Age at Measles1 Immunization by Lockdown Exposure 6
Figure S4: Probability of Completed First-Year Immunizations at the time of Survey, by Cohort 7
Table S7: Probability of Completed First-Year Immunizations at the time of Survey, by Cohort 7
Table S8: Completed First-Year Immunizations from Immunization Card and Parent Reports by Lockdown Exposure 8
Table S9: Difference in Early Immunizations by Lockdown Exposure 8
Table S10: Difference in Completed First-Year Immunization Status by Lockdown Exposure, Youngest Child per Household Only 9
Table S11: Changes in Completed Key First-Year Immunization Status Across Exposure Groups by Subgroup 9

A. HMIS Analysis

To supplement our survey results with data from across Rajasthan and all of India, we used publicly available immunization data from the government's Health Management Information System (HMIS), which collects monthly reports from the primary health system across the state. The collated data are publicly available online.[1,2] These data are based on paper records kept by frontline health workers and may have quality issues.[3] Nevertheless, they provide the most comprehensive measures of immunization activities in India and are used by policymakers for decision-making.

The outcomes available in the HMIS that we studied were immunization sessions, children that received each of the key first-year immunizations (BCG, Pentavalent1, Pentavalent2, Pentavalent3, Measles1), and children 9-11 months that were fully immunized. Figure S1 presents the monthly number of fully immunized children in Rajasthan from November 2019 to June 2020 (the last month of complete data), and for the same months in the previous 2 years. Numbers are relatively stable between November and February each year; but there is also evidence of seasonal trends. Therefore, to calculate changes over the lockdown period, we used data from November 2019 to June 2020 and November 2018 to June 2020 for the analysis. We pooled November to February as the reference and reported monthly percent changes relative to it. To account for historical trends, we also reported monthly changes in 2020 after adjusting for the change over the same period in the previous year: e.g., Change March2020 = 1 – (March2020/Reference2020)/(March2019/Reference2019). We also calculated changes separately for districts that were classified as COVID red zones between late April and mid-May and all other districts, because immunization outreach was suspended for longer in the former. To examine Rajasthan's experience relative to the rest of the country, we conducted the same exercise for fully immunized children aged 9-11 months for all of India, as well as for Bihar, Madhya Pradesh, and Uttar Pradesh.

Complete data are presented in supplement table S1 and historically adjusted changes in fully immunized children are presented in supplement figure S2. Monthly full immunizations in Rajasthan declined from 113,190 in the reference period to in 84,037 in March and 14,514 in April (-26% and -87% change), but numbers in May increased to 155,416 (37%). However, adjusting for historical trends increased the estimated decline to -45% in March and reduced the reversal in May to 23%. The remaining discussion focuses on adjusted estimates. Trends were similar across all key first-year immunizations, except for BCG, which experienced a smaller decline of 41% in April, possibly due to the government's policy to continue immunizations provided during births at health facilities through the lockdown. The rebound in May was substantially smaller in districts that had been classified as COVID red zones. Supply disruptions were a major explanatory factor, as monthly immunization sessions also decreased substantially in April. The recovery in May (23%) and June (-4%) was larger for Rajasthan than for India (-23% and -31%), suggesting that Rajasthan was among the more successful states in resuming immunization activities soon after the lockdown was eased.

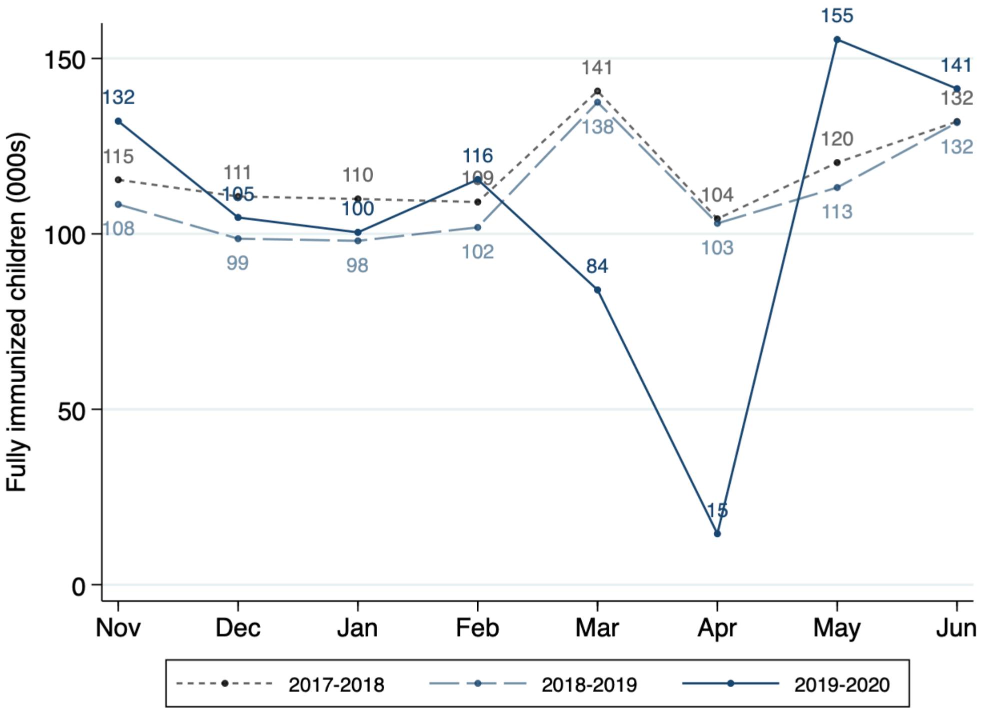

Figure S1: Monthly HMIS Trends of Fully Immunized Children Aged 9-11 Months in Rajasthan
The figure presents the monthly number of fully immunized children aged 9-11 months between November and June for 2020 and two previous years in Rajasthan as reported in the HMIS data.

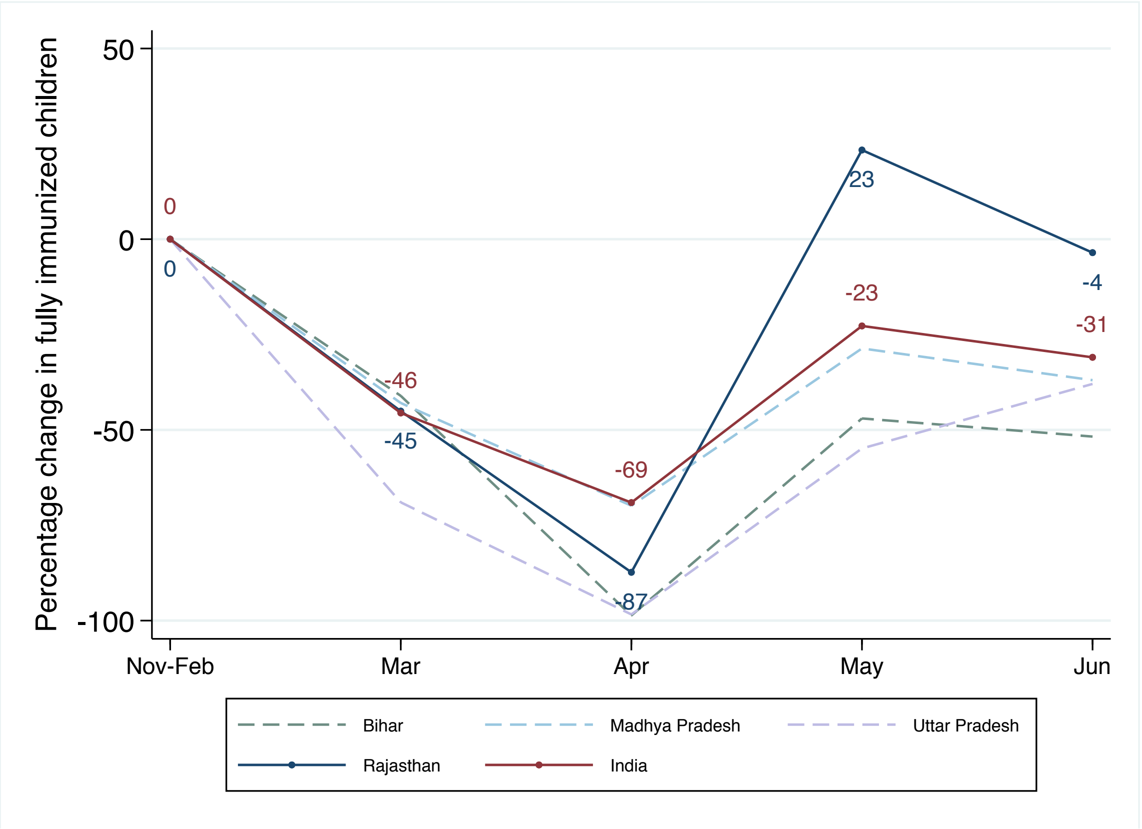

Figure S2: HMIS Changes in Monthly Fully Immunized Children Aged 9-11 Months in 2020
The graph presents monthly changes in fully immunized children 9-11 months between March and June 2020 relative to the November 2019 to February 2020 reference period, adjusting for changes over the same period in the previous year. The blue solid line is changes in Rajasthan. For comparison, the graph presents changes in all of India (red solid line), as well as Bihar, Madhya Pradesh, and Uttar Pradesh (dashed lines). Immunizations in Rajasthan dropped by 45% in March relative to pre-lockdown levels and by 87% in April, but increased by 23% in May. Both the decline and rebound were larger in Rajasthan than those in the all of India.

PANEL A: NOVEMBER 2019 TO JUNE 2020																
	**Nov-Feb Mean**	**March**			**April**			**May**			**June**			**March-June Mean**		
	Number	Number	% Ref	% Adj	Number	% Ref	% Adj	Number	% Ref	% Adj	Number	% Ref	% Adj	Number	% Ref	% Adj
**RAJASTHAN**																
Fully Immunized 9-11mo	113,190	84,037	-26	-45	14,514	-87	-87	155,416	37	23	141,389	25	-4	98,839	-13	-28
Immunization sessions	65,570	51,934	-21	-20	7,433	-89	-89	55,884	-15	-15	62,331	-5	-5	44,396	-32	-32
BCG	119,352	98,716	-17	-21	55,249	-54	-41	104,257	-13	1	103,417	-13	-8	90,410	-24	-17
Pentavalent1	127,436	88,930	-30	-26	16,167	-87	-84	140,996	11	43	113,771	-11	11	89,966	-29	-14
Pentavalent2	125,604	95,264	-24	-27	13,394	-89	-87	97,614	-22	-5	133,141	6	33	84,853	-32	-21
Pentavalent3	123,632	95,864	-22	-30	13,159	-89	-88	101,468	-18	-5	103,983	-16	-2	78,618	-36	-31
Measles1	119,214	86,600	-27	-46	15,251	-87	-87	161,833	36	22	147,304	24	-4	102,747	-14	-29
**COVID Red Zone**																
Fully Immunized 9-11mo	28,795	20,236	-30	-44	2,315	-92	-92	34,062	18	-0	34,411	20	-12	22,756	-21	-37
Immunization sessions	16,438	13,388	-19	-18	1,620	-90	-90	12,411	-25	-26	14,399	-12	-12	10,454	-36	-37
**COVID Other Zone**																
Fully Immunized 9-11mo	84,395	63,801	-24	-45	12,199	-86	-86	121,354	44	32	106,978	27	-0	76,083	-10	-25
Immunization sessions	49,132	38,546	-22	-20	5,813	-88	-88	43,473	-12	-11	47,932	-2	-2	33,941	-31	-30
**INDIA**																
Fully Immunized 9-11mo	2,059,236	1,805,660	-12	-46	664,406	-68	-69	1,880,668	-9	-23	1,822,266	-12	-31	1,543,250	-25	-42
Immunization sessions	1,081,912	981,357	-9	-14	396,937	-63	-62	835,248	-23	-27	842,482	-22	-26	764,006	-29	-32

PANEL B: NOVEMBER 2018 TO JUNE 2019											
	**Nov-Feb Mean**	**March**		**April**		**May**		**June**		**March-June Mean**	
	Number	Number	% Ref	Number	% Ref	Number	% Ref	Number	% Ref	Number	% Ref
**RAJASTHAN**											
Fully Immunized 9-11mo	101,733	137,524	35	103,000	1	113,214	11	131,732	29	121,368	19
Immunization sessions	65,208	64,258	-1	65,270	0	65,138	-0	65,082	-0	64,937	-0
BCG	118,575	124,692	5	93,642	-21	102,086	-14	112,112	-5	108,133	-9
Pentavalent1	128,418	120,833	-6	103,202	-20	99,142	-23	103,019	-20	106,549	-17
Pentavalent2	125,591	130,483	4	104,631	-17	102,496	-18	99,761	-21	109,343	-13
Pentavalent3	121,002	134,112	11	110,575	-9	104,963	-13	103,874	-14	113,381	-6
Measles1	108,569	146,556	35	109,168	1	120,335	11	139,581	29	128,910	19
**COVID Red Zone**											
Fully Immunized 9-11mo	25,607	32,159	26	25,386	-1	30,385	19	34,936	36	30,716	20
Immunization sessions	15,863	15,843	-0	15,841	-0	16,144	2	15,875	0	15,926	0
**COVID Other Zone**											
Fully Immunized 9-11mo	76,126	105,365	38	77,614	2	82,829	9	96,796	27	90,651	19
Immunization sessions	49,346	48,415	-2	49,429	0	48,994	-1	49,207	-0	49,011	-1
**INDIA**											
Fully Immunized 9-11mo	1,657,961	2,670,398	61	1,729,129	4	1,959,982	18	2,125,477	28	2,121,246	28
Immunization sessions	985,967	1,045,832	6	953,180	-3	1,045,400	6	1,037,008	5	1,020,355	3

Table S1: HMIS Monthly Immunization Trends in Rajasthan and India
The table presents immunization trends from the government's HMIS data for November 2019 to June 2020 in Panel A and November 2018 to June 2019 in Panel B for comparison. The monthly average in the November to February period is the reference period in each year. The table presents monthly numbers, the percentage change relative to the reference mean for the same year (% Ref), and the historically adjusted percentage change in 2020 that accounts for the change over the same period in the previous year (% Adj).

B. Survey Analysis

	Percent	Count	Observations
Sampled households		5,102	5,102
Reached by phone	0.670	3,418	5,102
Reached and eligible children confirmed	0.441	2,248	5,102
Consented to survey	0.926	2,081	2,248
Surveyed households			2,081
Children in Study			2,144

Table S2: Survey Status
This table presents complete statistics on the full sample of households sampled. The primary reasons for being unable to reach a household were that the phone number was invalid, outside coverage, or switched off, which was most likely due to households changing their phone numbers over time or possibly to numbers being entered incorrectly in the insurance records. Reached households which confirmed having at least one child aged 12-month-old or more living in the household were eligible for the survey. Children in study are the children aged at least 12 months old for whom we collected immunization data.

	(1) Not Surveyed	(2) Surveyed	(3) Overall	(4) (1) vs. (2), p-value
Age of mother at delivery	25.86	25.62	25.76	0.11
	(0.10)	(0.11)	(0.07)	
Residence village population	3974.52	4157.70	4048.92	0.76
	(362.99)	(505.71)	(297.71)	
Residence village low-caste share	0.37	0.34	0.36	0.00
	(0.01)	(0.01)	(0.00)	
Village has a public health facility	0.69	0.67	0.68	0.21
	(0.01)	(0.01)	(0.01)	
Village distance to nearest town	26.25	26.85	26.50	0.39
	(0.44)	(0.54)	(0.34)	
Village distance to disctrict HQ	59.36	60.13	59.68	0.51
	(0.74)	(0.87)	(0.56)	
COVID red zone	0.20	0.19	0.20	0.73
	(0.01)	(0.01)	(0.01)	
Observations	3,021	2,081	5,102	

Table S3: Household Characteristics by Survey Status
The table compares pre-survey characteristics of households that were sampled but not surveyed and those successfully surveyed. Columns 1-3 report group means and standard errors in parentheses. Column 4 reports the p-values from t-tests of the difference in means of each variable across surveyed and not surveyed households. Since survey data are unavailable for sampled but unreached households, we compare age of the mother at delivery, which is available in the insurance administrative claims data. We also obtain the household's residence village location from the claims data and link it to the 2011 Population Census, to allow us to compare household residence characteristics. Sampled households that were successfully reached for survey were statistically similar to sampled but unreached households in terms

	(1) Parents Report	(2) Immunization Card	(3) Overall	(4) (1) vs. (2), p-value
Male	0.55	0.51	0.52	0.09
	(0.02)	(0.01)	(0.01)	
Low caste	0.24	0.27	0.26	0.07
	(0.01)	(0.01)	(0.01)	
Low asset	0.55	0.47	0.50	0.00
	(0.02)	(0.01)	(0.01)	
Low parent education	0.55	0.53	0.54	0.40
	(0.02)	(0.01)	(0.01)	
COVID red zone	0.19	0.19	0.19	0.95
	(0.01)	(0.01)	(0.01)	
Observations	857	1287	2144	
Proportion	0.40	0.60	1.00	

Table S4: Characteristics of Children With and Without an Immunization Card
The table compares characteristics of children with an immunization card and those without one, where full immunization status was based on parent reports. Columns 1-3 report group means and standard errors in parentheses. Column 4 reports the p-values from pairwise t-tests of the differences of the means of each variable across the two sub-groups.

	(1) Unexposed	(2) Partially exposed	(3) Heavily exposed	(4) Post-exposure	(5) (1) vs. (2), p-value	(6) (1) vs. (3), p-value	(7) (1) vs. (4), p-value	(8) (2) vs. (3), p-value	(9) (2) vs. (4), p-value	(10) (3) vs. (4), p-value
Male	0.54	0.53	0.50	0.55	0.64	0.20	0.91	0.35	0.65	0.30
	(0.02)	(0.02)	(0.02)	(0.04)						
Has immunization card	0.61	0.62	0.58	0.61	0.82	0.28	0.95	0.13	0.91	0.40
	(0.02)	(0.02)	(0.02)	(0.04)						
Low asset	0.48	0.49	0.51	0.51	0.60	0.23	0.42	0.45	0.64	1.00
	(0.02)	(0.02)	(0.02)	(0.04)						
Low caste	0.23	0.26	0.28	0.24	0.15	0.04	0.69	0.49	0.53	0.29
	(0.02)	(0.02)	(0.02)	(0.03)						
Low parent education	0.53	0.55	0.53	0.57	0.52	0.96	0.39	0.41	0.65	0.34
	(0.02)	(0.02)	(0.02)	(0.04)						
COVID red zone	0.20	0.19	0.20	0.19	0.62	0.91	0.83	0.66	0.89	0.88
	(0.02)	(0.01)	(0.01)	(0.03)						
Observations	443	722	796	183						
Proportion	0.21	0.34	0.37	0.09						

Table S5: Child Characteristics Across Exposure Groups
The table compares characteristics of children classified into the four exposure groups. Columns 1-4 report group means and standard errors in parentheses. Columns 5-10 report the p-values from pairwise t-tests of the differences of the means of each variable across exposure groups.

	Unadjusted						Adjusted					
	Measles1 at/before 9mo			Measles1 10-12mo			Measles1 at/before 9mo			Measles1 10-12mo		
	OR	p-value	95% Confidence Interval	OR	p-value	95% Confidence Interval	OR	p-value	95% Confidence Interval	OR	p-value	95% Confidence Interval
Unexposed	Ref			Ref			Ref			Ref		
Partially exposed	0.882	0.532	[0.596,1.306]	1.227	0.305	[0.830,1.813]	0.880	0.523	[0.594,1.304]	1.227	0.306	[0.829,1.816]
Heavily exposed	0.547	0.003	[0.366,0.819]	1.765	0.004	[1.202,2.592]	0.550	0.004	[0.367,0.824]	1.761	0.004	[1.196,2.591]
Post-exposure	0.964	0.882	[0.590,1.574]	1.447	0.135	[0.891,2.348]	0.986	0.955	[0.602,1.615]	1.403	0.174	[0.861,2.284]
Observations	865			865			865			865		

Table S6: Measles1 Immunization at or before 9 months and between 10-12 months by Lockdown Exposure
Odds ratios from logistic regressions of timeliness of Measles1 outcomes on indicators for exposure group, with unexposed children that turned 12 months old before March 2020 as the reference group. The sample is restricted to children with an immunization card and date of Measles1 recorded, if received. Adjusted regressions include indicators for survey month, low assets, and low caste as controls. OR = odds ratio

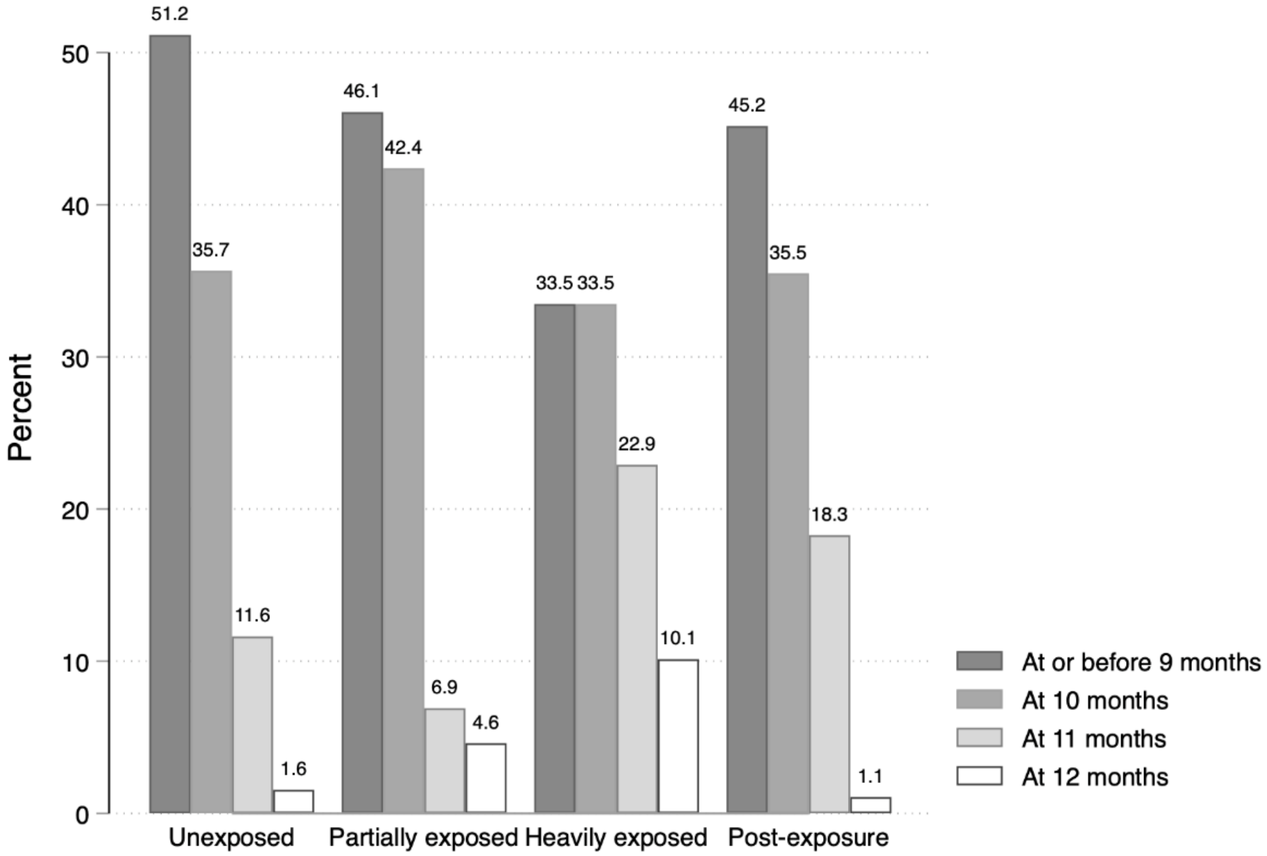

Figure S3: Age at Measles1 Immunization by Lockdown Exposure
The figure presents the breakdown of age at which children received their Measles1 immunization by lockdown exposure group, among children with any Measles1 on their immunization card.

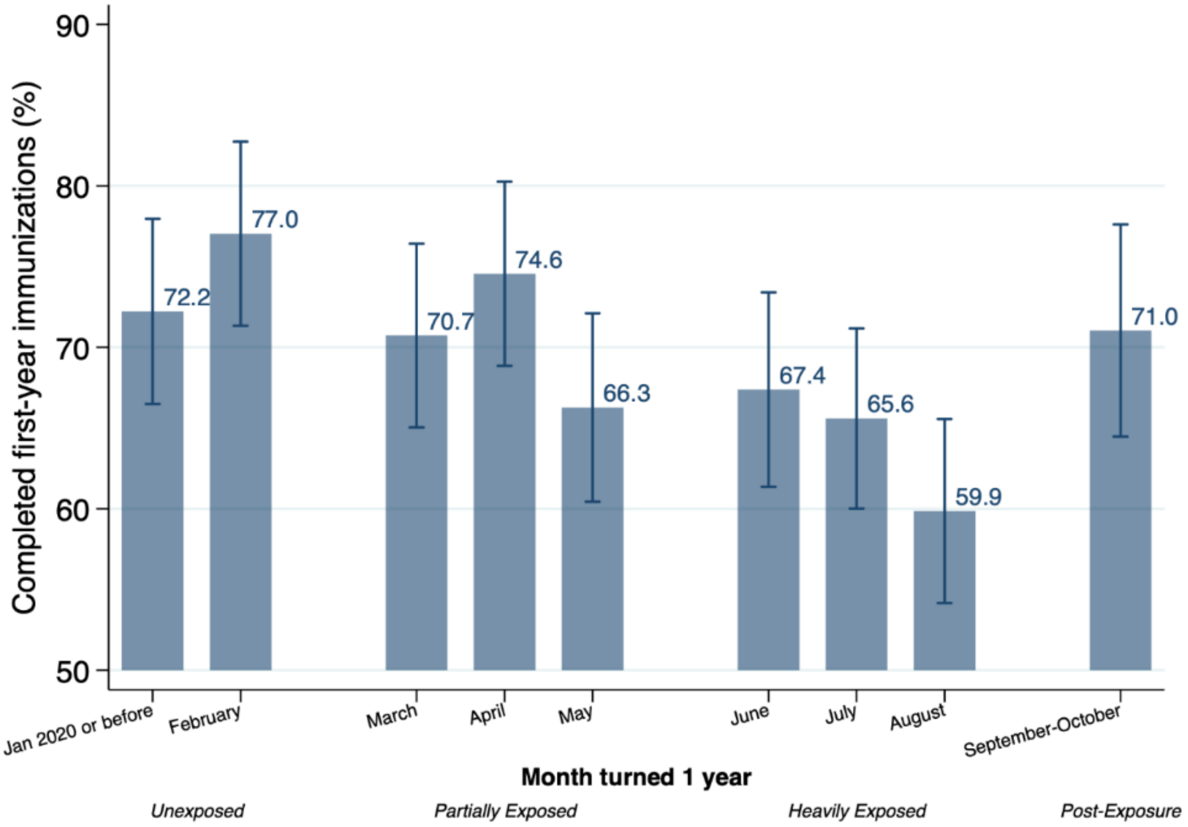

Figure S4: Probability of Completed First-Year Immunizations at the time of Survey, by Cohort
The figure presents unadjusted predicted probabilities of having received all key first-year immunizations by age cohort with 95% confidence intervals. The outcome is equal to one if the child received Pentavalent1, Pentavalent2, Pentavalent3, and Measles1 on the immunization card or at least 4 injectable immunizations per parent reports, if the immunization card was unavailable. Numbers underlying the figure are in table S7.

	Observations	Probability	95% Confidence Interval
Jan 2020 or before	234	0.722	[0.665,0.780]
February	209	0.770	[0.713,0.827]
March	246	0.707	[0.650,0.764]
April	224	0.746	[0.688,0.803]
May	252	0.663	[0.604,0.721]
June	233	0.674	[0.614,0.734]
July	279	0.656	[0.600,0.712]
August	284	0.599	[0.542,0.656]
September-October	183	0.710	[0.645,0.776]
Observations	2,144		

Table S7: Probability of Completed First-Year Immunizations at the time of Survey, by Cohort
The table presents the numbers underlying Figure S4.

	Unadjusted						Adjusted					
**Panel A: Probability of Completed First-Year Immunizations**												
	From Immunization Card		From Parent Reports				From Immunization Card		From Parent Reports			
	PR	95% Confidence Interval	PR	95% Confidence Interval			PR	95% Confidence Interval	PR	95% Confidence Interval		
Unexposed	0.841	[0.797,0.884]	0.644	[0.578,0.710]			0.840	[0.796,0.883]	0.644	[0.579,0.709]		
Partially exposed	0.784	[0.746,0.822]	0.623	[0.570,0.677]			0.783	[0.745,0.821]	0.623	[0.570,0.676]		
Heavily exposed	0.748	[0.708,0.788]	0.531	[0.480,0.582]			0.748	[0.709,0.788]	0.535	[0.485,0.586]		
Post-exposure	0.786	[0.710,0.862]	0.610	[0.501,0.719]			0.791	[0.715,0.867]	0.590	[0.478,0.702]		
**Panel B: Change in Completed First-Year Immunizations Relative to Unexposed Children**												
	From Immunization Card			From Parent Reports			From Immunization Card			From Parent Reports		
	OR	p-value	95% Confidence Interval	OR	p-value	95% Confidence Interval	OR	p-value	95% Confidence Interval	OR	p-value	95% Confidence Interval
Unexposed	Ref			Ref			Ref			Ref		
Partially exposed	0.689	0.065	[0.463,1.024]	0.917	0.643	[0.635,1.323]	0.687	0.065	[0.461,1.023]	0.911	0.623	[0.628,1.321]
Heavily exposed	0.562	0.004	[0.381,0.828]	0.627	0.010	[0.441,0.892]	0.564	0.004	[0.382,0.833]	0.630	0.011	[0.441,0.901]
Post-exposure	0.695	0.199	[0.398,1.212]	0.868	0.607	[0.505,1.490]	0.721	0.261	[0.407,1.275]	0.791	0.412	[0.451,1.386]
Observations	1,287			966			1,287			966		

Table S8: Completed First-Year Immunizations from Immunization Card and Parent Reports by Lockdown Exposure

In the main analysis we combined children with key first-year immunization status obtained from the immunization card and from parent reports. The table reports separate estimates of first-year immunization status separately for each data source. Panel A presents the predicted probabilities of having received all key first-year immunizations by lockdown exposure group and data source. The outcome is equal to one if the child received Pentavalent1, Pentavalent2, Pentavalent3, and Measles1 on the immunization card or at least 4 injectable immunizations per parent reports, if the immunization card was unavailable. Panel B presents odds ratios with 95% confidence intervals of the same outcome on indicators for exposure group, with unexposed children that turned 12 months old before March 2020 as the reference group. Adjusted regressions include indicators for survey month, low assets, and low caste as controls. PR = probability OR = odds ratio

	Unadjusted						Adjusted					
	Pentavalent1			Pentavalent2			Pentavalent1			Pentavalent2		
	OR	p-value	95% Confidence Interval	OR	p-value	95% Confidence Interval	OR	p-value	95% Confidence Interval	OR	p-value	95% Confidence Interval
Unexposed	Ref			Ref			Ref			Ref		
Partially exposed	0.815	0.558	[0.411,1.615]	0.753	0.411	[0.383,1.481]	0.832	0.599	[0.418,1.653]	0.750	0.407	[0.380,1.480]
Heavily exposed	0.780	0.472	[0.397,1.534]	0.725	0.346	[0.371,1.415]	0.801	0.521	[0.406,1.580]	0.728	0.355	[0.372,1.427]
Post-exposure	1.082	0.883	[0.377,3.111]	0.658	0.366	[0.265,1.633]	1.001	0.999	[0.339,2.956]	0.642	0.354	[0.252,1.639]
Observations	1,287			1,287			1,287			1,287		

Table S9: Difference in Early Immunizations by Lockdown Exposure

Odds ratios with 95% confidence intervals from logistic regressions of binary measures for receipt of key first-9 months immunizations on indicators for exposure group. Unexposed children that turned 12 months old before March 2020 are the reference group. The outcomes are respectively equal to one if the child has received BCG, Pentavalent1, and Pentavalent2 on the immunization card. The sample is restricted to children whose outcomes were measured from the immunization card. Adjusted regressions include indicators for survey month, low assets, and low caste as controls. OR = odds ratio

	Unadjusted			Adjusted		
**Panel A: Probability of Completed First-Year Immunizations**						
	PR		95% Confidence Interval	PR		95% Confidence Interval
Unexposed	0.744		[0.702,0.785]	0.739		[0.699,0.780]
Partially exposed	0.706		[0.672,0.740]	0.702		[0.669,0.734]
Heavily exposed	0.641		[0.607,0.675]	0.649		[0.617,0.681]
Post-exposure	0.709		[0.643,0.776]	0.705		[0.639,0.772]
**Panel B: Change in Completed First-Year Immunizations Relative to Unexposed Children**						
	OR	p-value	95% Confidence Interval	OR	p-value	95% Confidence Interval
Unexposed	Ref			Ref		
Partially exposed	0.828	0.173	[0.631,1.087]	0.818	0.161	[0.617,1.083]
Heavily exposed	0.616	0.000	[0.474,0.801]	0.631	0.001	[0.481,0.828]
Post-exposure	0.842	0.388	[0.571,1.243]	0.833	0.385	[0.552,1.258]
Observations	2,081			2,081		

Table S10: Difference in Completed First-Year Immunization Status by Lockdown Exposure: One Child Per Household Only

The table presents unadjusted and covariate-adjusted predicted probabilities of having received all key first-year immunizations by exposure group in Panel A. The unadjusted probabilities are the same as presented in Figure 2. Panel B presents odds ratios from logistic regressions of the same binary outcome on indicators for exposure group, with unexposed children that turned 12 months old before March 2020 as the reference group. The outcome is equal to one if the child received Pentavalent1, Pentavalent2, Pentavalent3, and Measles1 on the immunization card or at least 4 injectable immunizations per parent reports, if the immunization card was unavailable. Adjusted regressions include indicators for survey month, low assets, low caste, and whether the child had an immunization card as controls. The table is identical to Table 2 in the paper, but here we restrict the sample to one child per household; in the case of 63 households with more than child, we only include the youngest eligible child. PR = probability OR = odds ratio

	(1)	(2)	(3)	(4)	(5)	(6)	(7)
	**Probability of Completed First-Year Immunizations (%)**			**PP Difference Heavily Exposed - Unexposed (95% CI)**	**p-value**	**PP Subgroup Difference (95% CI)**	**p-value**
	**Unexposed**	**Heavily Exposed**	**Post-Exposure**				
**Female**	73.4	67.5	75.3	-5.8 (-13.2 to 1.6)	0.123		
**Male**	75.1	61.8	66.9	-13.4 (-20.5 to -6.3)	0.000	-7.5 (-17.7 to 2.8)	0.152
**High assets**	77.1	70.4	74.1	-6.5 (-13.3 to 0.3)	0.061		
**Low assets**	71.5	59.3	67.0	-12.4 (-19.9 to -4.8)	0.001	-5.6 (-15.8 to 4.6)	0.284
**High parent education**	79.0	73.2	67.8	-5.8 (-12.6 to 1.1)	0.101		
**Low parent education**	70.1	57.1	72.7	-13.0 (-20.3 to -5.7)	0.000	-7.2 (-17.3 to 2.8)	0.160
**High caste**	74.8	66.8	69.2	-8.0 (-13.9 to -2.2)	0.007		
**Low caste**	72.6	58.9	75.5	-13.5 (-23.9 to -3.0)	0.011	-5.6 (-17.7 to 6.4)	0.359
**COVID other zones**	73.0	65.3	72.7	-7.7 (-13.4 to -2.0)	0.009		
**COVID red zones**	79.9	62.0	62.0	-17.9 (-29.0 to -6.8)	0.002	-10.2 (-22.7 to 2.3)	0.109

Table S11: Changes in Completed Key First-Year Immunization Status Across Exposure Groups by Subgroup

Columns 1-3 present the predicted probabilities of having received all key first-year immunizations for each exposure group by subgroup. Column 4 presents the percentage point difference in probability between the heavily exposed and unexposed groups by subgroup and Column 5 presents the p-value for this comparison. Column 6 presents the percentage point difference in subgroup differences and Column 7 presents the p-value for this comparison. 95% Confidence Intervals are included in parenthesis. Estimates include controls for survey month and whether the child had an immunization card.

REFERENCES

1 Asher S, Novosad P. Development Data Lab COVID India. [Internet] Available: https://www.dropbox.com/sh/y949ncp39towulf/AABs8_dECTzr38GdS7BneTH7a?dl=0 [Accessed 10 October 2020].

2 National Rural Health Mission. HMIS web portal [Internet]. Available: https://nrhm-mis.nic.in/hmisreports/frmstandard_reports.aspx [Accessed 10 October 2020].

3 Sharma A, Rana SK, Prinja S, et al. Quality of Health Management Information System for Maternal & Child Health Care in Haryana State, India. *PLoS One* 2016;**11**:1–11. doi:10.1371/journal.pone.0148449.
